# Differences in Organic Solute and Metabolites of *Leymus chinensis* in Response to Different Intensities of Salt and Alkali Stress

**DOI:** 10.3390/plants12091916

**Published:** 2023-05-08

**Authors:** Ge Yan, Yujie Shi, Chunsheng Mu, Junfeng Wang

**Affiliations:** Key Laboratory of Vegetation Ecology, Ministry of Education, Institute of Grassland Science, School of Life Sciences, Northeast Normal University, No. 5268 Renmin Street, Changchun 130024, China

**Keywords:** physiological response, soluble sugar, amino acid, betaine, organic acid, energy supply

## Abstract

To explore differences in the physiological metabolic response mechanisms of grassland perennial plants to different intensities of salt–alkali stress, we employed GC-MS to identify the metabolome of perennial rhizome-saline-tolerant *Leymus chinensis* (*L. chinensis*). *L. chinensis* reduced stress damage by accumulating osmotic solutes during salt–alkali stress, although the types of accumulated solutes varied with stress and concentration gradients. Soluble sugars increased only under mild salt–alkali stress. Under salt and mild alkali stress, amino acids increased. Under severe salt–alkali stress, organic acids increased. Betaine increased as a typical osmolute under salt–alkali stress. Metabolic analysis identified 20 metabolites, including 4 amino acids, 6 sugars, and 10 organic acids. The majority of them increased in response to stress. Under mild salt stress, the metabolites included glycine and proline. Under mild alkali stress, they primarily consisted of sugars such as isomaltose and lactulose, whereas under severe salt–alkali stress, they primarily consisted of organic acids such as citric acid and isocitric acid. Pathway analysis showed that six pathways were affected. Glycine, serine, and threonine metabolism was affected under mild salt stress. Alanine, aspartate, and glutamate metabolism and butanota metabolism were affected under mild alkali stress, while energy metabolism pathways, such as the TCA cycle and glyoxylate and dicarboxylate metabolism, were affected under severe salt–alkali stress. The results indicate the importance of betaine in stress resistance and the significance of organic acid in severe salt stress, and they also demonstrate that energy supply was one of the key mechanisms in response to severe salt–alkali stress.

## 1. Introduction

Soil salinization has a detrimental effect on plant growth and agricultural production. At present, the world’s saline–alkali land area is roughly 10^9^ hm^2^ [1], including 20% of arable land and 50% of irrigated land [2]. Soil salinization has a negative effect on plants via the combination of salt and alkali stress. Salt stress causes excessive Na^+^ to enter cells, leading to ion toxicity; on the other hand, high ion concentrations outside cells increase cell water loss, resulting in physiological drought, also referred to as osmotic stress, all of which induce metabolic disorders in plants [3]. Alkali stress has a high pH on the basis of salt stress. As a result, besides osmotic stress and ion toxicity, it can adversely impact intracellular pH stability, ruin cell membrane integrity, limit photosynthesis, impede the accumulation of photosynthetic pigments, influence plant growth, and even cause death [3,4]. Salt stress and alkali stress are two different types of stress.

Under stress conditions, plants produce a variety of biological substances, including hundreds of metabolites, which play a significant role in the plant’s stress response. Plants can adapt to environmental changes by changing their physiology through metabolic changes [5]. Because metabolites are the end products of biological systems, changes in metabolic levels in organisms are expected to be connected with phenotypes. Numerous studies have confirmed that some metabolites, such as proline, betaine, organic acids, soluble sugars, nitrogen compounds, and polyols, are beneficial in improving plant salt tolerance [3,6,7]. Meanwhile, plants also need to accumulate specific metabolites to balance intracellular pH in addition to the metabolites listed above under alkali stress. Among these, organic acid accumulation is a form of plant adaptation to high pH; multiple studies have demonstrated that an increase in organic acid concentration improved plants’ alkali stress resistance [6,7]. Metabolite accumulation involves the activation of metabolic processes, and plants enhance metabolite accumulation by activating metabolic pathways, hence strengthening stress tolerance. A variety of studies have revealed that stimulating the synthesis pathways of the osmotic solutes mentioned above, such as amino acids, betaine, and soluble sugars, is critical for improving plant salt tolerance [2,6,7,8]. Energy supply pathways, such as β-oxidation, glycolysis, and the TCA cycle are typically important for enhancing plant alkali tolerance [9,10,11]. The unique metabolic response mechanism of plants to salt and alkali stress, however, varies by species. In addition, it has not been confirmed whether the physiological response mechanism of plants alters under different concentrations of salt and alkali stress.

*L. chinensis* is a salt- and alkali-tolerant perennial rhizome grass from the steppes of northern China. It has a high feeding value and can form a community in alkali areas of saline–alkali land. As a result, it is one of the most suitable grass species for saline–alkali land improvement [1,12]. Currently, researchers are investigating the response of *L. chinensis* to salt and alkali stress using transcriptome and proteome approaches [13,14], while the physiological response of *L. chinensis* to salt and alkali stress is currently being investigated only through multiple physiological experiments to study the accumulation of specific metabolites under salt and alkali stress. *L. chinensis* enhances salt tolerance predominantly by accumulating proline, betaine, and soluble sugar, and alkali tolerance primarily by accumulating organic acids (citric acid, malic acid, succinic acid, acetic acid, and oxalic acid) [12,15]. A few studies have used metabonomics to explore how *L. chinensis* could alleviate damage and adapt to stress by modulating its metabolic network. The booting stage is crucial for plant growth and development since plants absorb a great deal of water and nutrients during this period and are highly sensitive to changes in the external environment. Meanwhile, plant growth and development during this period will exert a noticeable effect on eventual production [16]. Under salt stress, studies on rice [17] and sweet sorghum [18] have revealed that the booting stage is the most susceptible period for these two crops. However, little research on the sensitivity of *L. chinensis* to salt and alkali stress during the booting stage has been reported. Therefore, we chose the booting stage of *L. chinensis* in this study and utilized gas chromatography–mass spectrometry (GC-MS) to explore the changes in organic solutes, metabolites, and metabolic pathways under salt and alkali stress at various concentrations. The objective of this study was to evaluate the differences in the organic solutes and metabolism of *L. chinensis* in response to salt and alkali stress, as well as different stress concentrations of the same type of stress, so as to gain insight into the internal physiological response and mediation mechanism. Our research fills a gap in the knowledge about the metabolic response of perennial grasses to varying concentration gradients of salt and alkali stress.

## 2. Results and Discussion

### 2.1. Changes in Growth Parameters of L. chinensis under Salt and Alkali Stress

**Figure 1:**
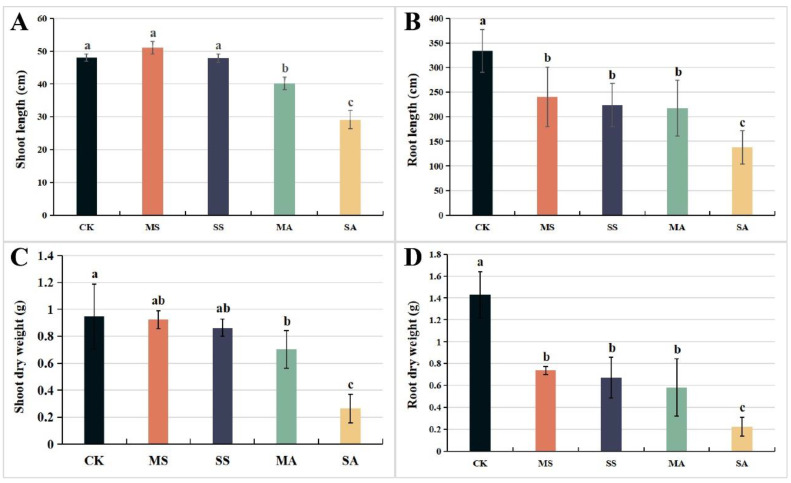
Changes in (**A**) shoot length, (**B**) root length, (**C**) shoot dry weight, and (**D**) root dry weight of *L. chinensis* under different concentrations of salt and alkali stress. Values represent the means of six replicates. Different letters indicate significant differences among different concentrations of salt and alkali stress (*p* < 0.05), while the same letters indicate no difference between different concentrations of salt and alkali stress. CK: control group; MS: 200 mmol/L NaCl treatment; SS: 400 mmol/L NaCl treatment; MA: 25 mmol/L Na_2_CO_3_ treatment; SA: 50 mmol/L Na_2_CO_3_ treatment.

Under mild and severe salt stress, there were no significant differences in the plant height and shoot dry weight, but they decreased significantly under mild and severe alkali stress. Especially under severe alkali stress, the plant height and shoot dry weight decreased by 39.3% and 72.2%, respectively, compared to the control (Figure 1A,C). Under different concentrations of salt stress and alkali stress, the root length and root dry weight decreased significantly, and the degree of decline increased with the increase in the two stress concentrations. Under severe salt and alkali stress, the root length and root dry weight decreased by 68.6% and 77.3%, respectively. (Figure 1B,D). This shows that alkali stress inhibited the growth of *L. chinensis* more than salt stress and damaged the root more than the shoot.

### 2.2. Changes in Organic Solutes under Salt and Alkali Stress

**Figure 2:**
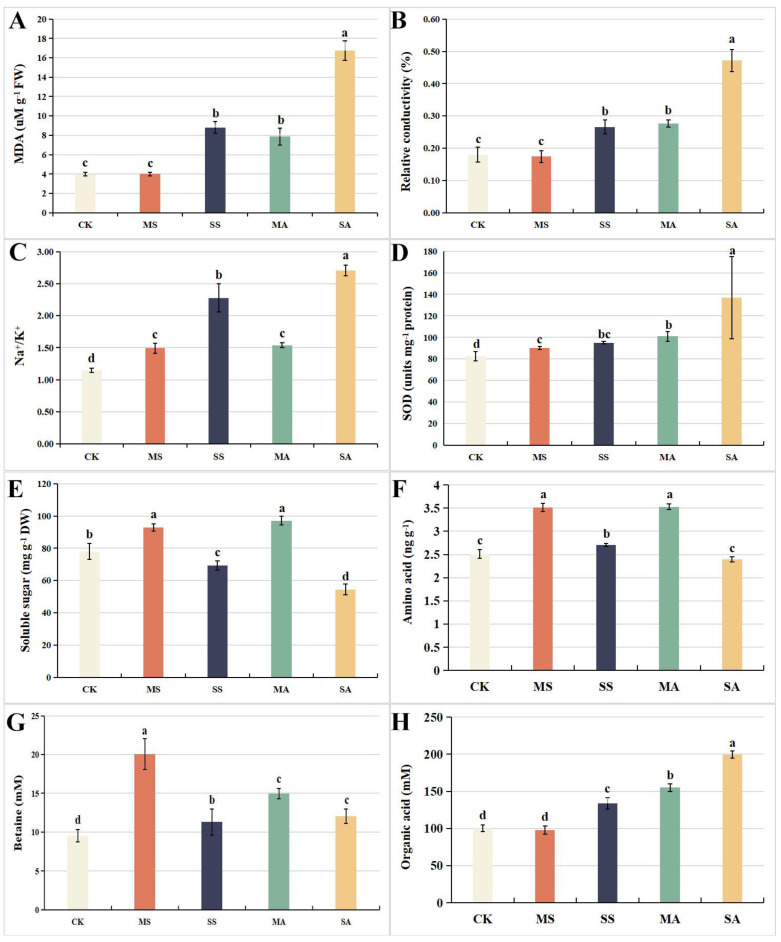
Changes in (**A**) MDA, (**B**) relative conductivity, (**C**) Na^+^/K^+^, (**D**) superoxide dismutase (SOD), (**E**) soluble sugars, (**F**) amino acids, (**G**) betaine, and (**H**) organic acids of *L. chinensis* under different concentrations of salt and alkali stress. Values represent the means of six replicates. Different letters indicate significant differences among different concentrations of salt and alkali stress (*p* < 0.05), while the same letters indicate no difference between different concentrations of salt and alkali stress. CK: control group; MS: 200 mmol/L NaCl treatment; SS: 400 mmol/L NaCl treatment; MA: 25 mmol/L Na_2_CO_3_ treatment; SA: 50 mmol/L Na_2_CO_3_ treatment.

Under mild salt stress, although the Na^+^/K^+^ and SOD increased significantly, the differences in the malondialdehyde (MDA) and relative conductivity were not significant. Under severe salt stress and alkali stress, the Na^+^/K^+^, SOD, malondialdehyde (MDA), and relative conductivity increased significantly with the increase in the stress concentration; the highest value was under severe alkali stress, which increased by 135.7%, 66.0%, 162.2%, and 320.8%, respectively, compared to the control (Figure 2A–C).

Previous studies have shown that salt and alkali stress can cause a considerable accumulation of Na^+^ and reactive oxygen species (ROS), resulting in ion toxicity and oxidative stress, as well as the destruction of cell membrane integrity, resulting in plant growth inhibition and even death [6,19]. The relative conductivity and MDA are important indicators for assessing the degree of cell membrane damage, and there are direct correlations between their levels and plant salt tolerance; they all increase significantly as the soil salinity and pH increase, especially under high−concentration saline−alkali stress [20,21]. Our findings are consistent with the previous research, and we discovered that the values of the three indicators under alkali stress were significantly higher than those under salt stress, indicating that the damaging effect of alkali stress on the *L. chinensis* cell membrane was greater than that of salt stress and that *L. chinensis* increased SOD synthesis to remove excess ROS, which could have been due to the simultaneous ion toxicity and high pH stress caused by alkali stress [22].

Under mild salt stress, the contents of soluble sugar, amino acid, and betaine increased significantly compared to the control, which increased by 19.0%, 39.9%, and 56.9%, respectively, while the organic acids had no significant difference. Under severe salt stress, although the contents of amino acids and betaine also increased significantly, the increase range was significantly smaller than that under mild salt stress, while the contents of organic acids increased significantly at this time. Under mild alkali stress, the contents of soluble sugar, amino acids, betaine, and organic acids all increased significantly. Under severe alkali stress, the contents of betaine also increased significantly, but the increase range was significantly smaller than that under mild alkali stress, while the contents of organic acid increased with the increase in alkali stress and reached its highest value under severe alkali stress, which was an increase of 99.3% compared to the control.

Plants can respond to salt and alkali stress by accumulating small-molecule osmotic solutes in order to reduce stress damage [6]. Previous research has revealed that when plants are subjected to salt and alkali stress, the concentrations of soluble sugars [14,15,21], organic acids [12,13,15,23,24], amino acids [6,25], and betaine [21] increase, which are the main solutes to resist stress. However, the solutes accumulated by plants in response to salt and alkali stress change depending on different plant species and stress concentrations.

### 2.3. Changes in Metabolites under Salt and Alkali Stress

**Figure 3:**
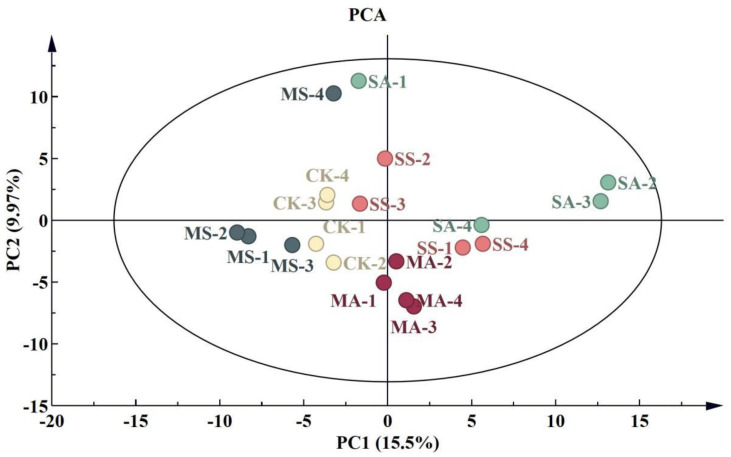
PCA analysis of the metabolic profiles of *L. chinensis* under salt and alkali stress. The number of repetitions was four. CK: control group; MS: 200 mmol/L NaCl treatment; SS: 400 mmol/L NaCl treatment; MA: 25 mmol/L Na_2_CO_3_ treatment; SA: 50 mmol/L Na_2_CO_3_ treatment.

A total of 228 original peaks were obtained in the leaf samples of *L. chinensis* seedlings by GC-MS detection (Supplementary Table S1). The results of the PCA show that the samples in different treatment groups were clearly distinguished between the two principal components, and the different samples in the same treatment group clustered well. A total of 25.47% of the variables in the metabolites could be explained by the two principal components (Figure 3). The metabolites with the highest contribution to PC1 were mainly organic acids and soluble sugars, including citric acid, malonic acid, lactic acid, itaconic acid, lactulose, and myo-inositol. The metabolites with the highest contribution to PC2 were mainly organic acids, including lactic acid, mucic acid, quinic acid, malonic acid, and malic acid (Supplementary Table S2).

**Figure 4:**
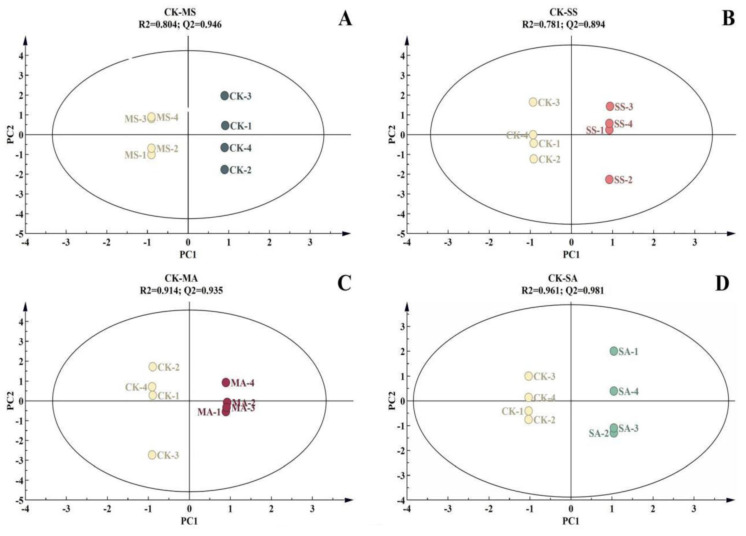
OPLS−DA analysis of metabolic profiles of *L. chinensis* leaves under salt and alkali stress at different concentrations of salt and alkali stress. The number of repetitions was four. (**A**) CK−MS, (**B**) CK−SS, (**C**) CK−MA, and (**D**) CK−SA CK: control group; MS: 200 mmol/L NaCl treatment; SS: 400 mmol/L NaCl treatment; MA: 25 mmol/L Na_2_CO_3_ treatment; SA: 50 mmol/L Na_2_CO_3_ treatment.

The data for different concentrations of the salt and alkali treatments were further analyzed by OPLS-DA. R^2^ and Q^2^ represent the fitting degree and predictive ability of the model, respectively. The results show that the R^2^ values of the four treatment models, including CK−MS (Figure 4A), CK−SS (Figure 4B), CK−MA (Figure 4C), and CK−SA (Figure 4D), were all greater than 0.7, and the Q^2^ values were greater than 0.9, indicating that the fitting degree and predictive ability of the model were good, further indicating that there were significant differences in the samples among different treatments.

Based on the criteria of vip > 1 and *p* < 0.05, a total of 20 differential metabolites were screened out, including 4 amino acids, 6 soluble sugars, and 10 organic acids. Among CK−MS, CK−SS, CK−MA, and CK−SA, 2, 6, 7, and 10 differential metabolites were identified, respectively. Among the differential metabolites, the contents of GABA decreased significantly under severe salt stress and alkali stress, and the contents of mucic acid and maltotriose decreased significantly under mild and severe alkali stress, respectively. Among them, the fold change of GABA was less than −27, and the contents of the remaining 17 differential metabolites increased significantly, among which the fold changes of proline, glycine, isocitric acid, and isomaltose were greater than 29.

**Figure 5:**
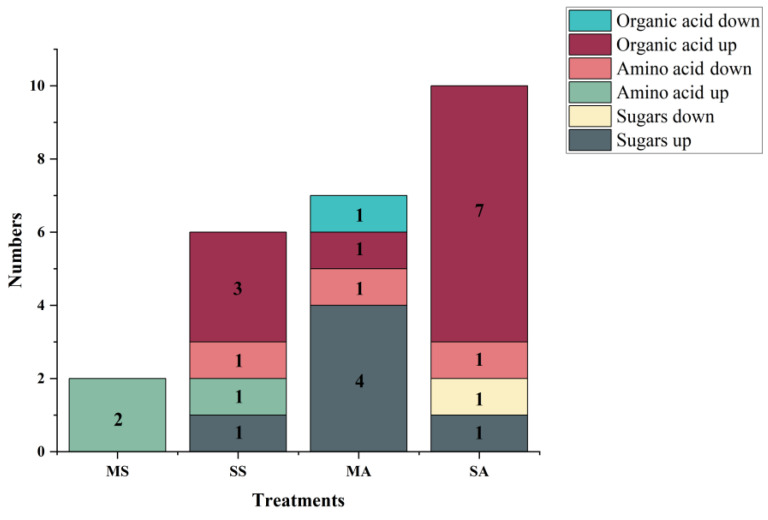
The numbers of upregulated and downregulated differential metabolites in *L. chinensis* under salt and alkali stress. The number in each box represents the number of metabolites. MS: 200 mmol/L NaCl treatment; SS: 400 mmol/L NaCl treatment; MA: 25 mmol/L Na_2_CO_3_ treatment; SA: 50 mmol/L Na_2_CO_3_ treatment.

**Figure 6:**
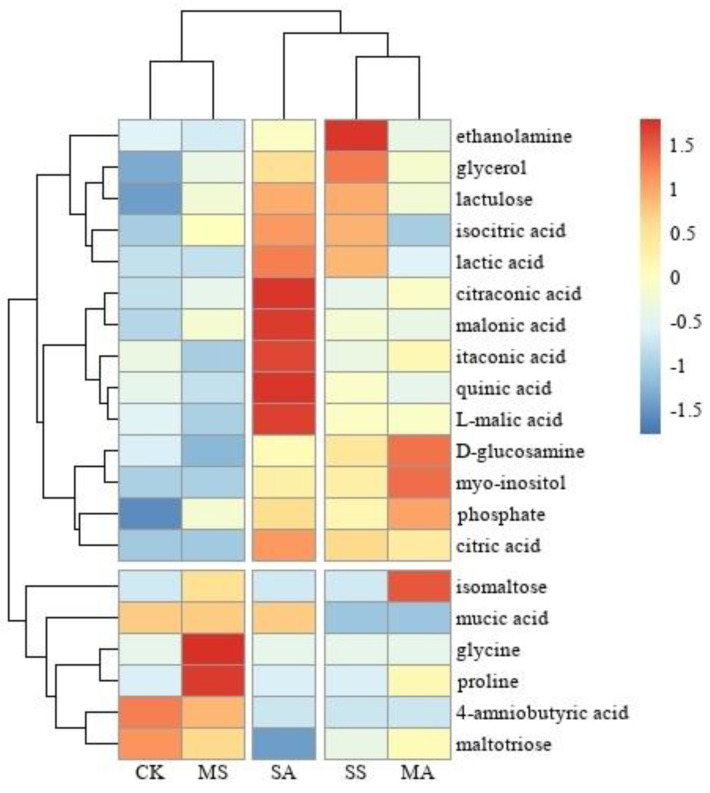
Cluster heat map analysis of differential metabolites of *L. chinensis* under salt and alkali stress. MS: 200 mmol/L NaCl treatment; SS: 400 mmol/L NaCl treatment; MA: 25 mmol/L Na_2_CO_3_ treatment; SA: 50 mmol/L Na_2_CO_3_ treatment.

Under mild salt stress, the contents of two amino acids, glycine and proline, increased significantly, and the fold change of isomaltose was greater than 28, showing an obvious increasing trend. Under severe salt stress, the contents of ethanolamine, glycerol, and three organic acids, including citric acid, isocitric acid, and lactic acid, increased significantly. Under mild alkali stress, the contents of phosphate and four soluble sugars, including glucosamine, isomaltose, lactulose, and myo-inositol, increased significantly, and the fold change of proline was greater than 28, which also showed a significant increasing trend. Under severe alkali stress, glycerol and seven organic acids, including quinic acid, citraconic acid, citric acid, isocitric acid, itaconic acid, malic acid, and malonic acid, increased significantly (Figure 5 and Figure 6 and Supplementary Table S3).

Previous studies have demonstrated that the contents of amino acids and soluble sugars increase with increases in salt and alkali stress, but this study discovered that the contents of glycine and proline increased significantly only under mild salt stress, while the contents of soluble sugars, such as glucosamine, isomaltose, lactulose, and myo-inositol, increased significantly only under mild alkali stress, with no significant accumulation of the two under severe salt and alkali stress. Zhang et al. [26] investigated the response of *Puccinellia tenuiflora* to salt and alkali stress and discovered that the amino acid mass fraction (mg/g) increased first and then decreased as the stress concentration increased. Adams [27] discovered a similar trend in his study of the soluble sugar content of *Mesembryanthemum crystallinum* under salt stress. Some researchers have suggested that changes in the amino acid and soluble sugar contents can be utilized to assess plant resistance to salt and alkali stress [28,29].

Plants accumulate organic acids to adapt to the high pH environment caused by alkali stress. Alkali stress causes *L. chinensis* to accumulate organic acids, such as citric acid, malic acid, and succinic acid [12,15,23,24]. The results of this study indicate that the contents of citric acid and malic acid in *L. chinensis* significantly increased under severe alkali stress. It was also discovered that the contents of five organic acids, including isocitric acid, quinic acid, and malonic acid, also significantly increased under severe alkali stress. This, combined with the findings that the contents of organic acid increased significantly under severe alkali stress, demonstrates the importance of organic acid in balancing the intracellular pH of *L. chinensis* in response to alkali stress. Large increases in the levels of citric acid, isocitric acid, and lactic acid were detected under severe salt stress, in addition to alkali stress. Since salt stress did not generate a high pH environment but caused an imbalance of intracellular and intracellular osmotic pressure [6,30], organic acids may be primarily involved in the regulation of intracellular osmotic pressure balance, contrary to previous studies on *L. chinensis*, which indicated that organic acids as osmotic solutes were less involved in osmoregulation under salt stress [13].

### 2.4. Changes in Metabolic Pathways under Salt and Alkali Stress

**Figure 7:**
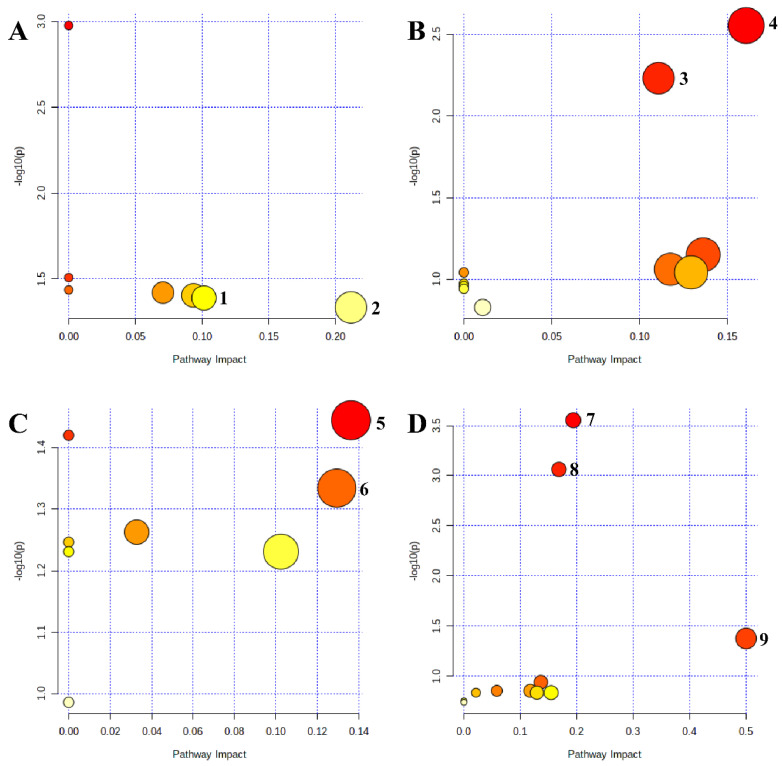
Enrichment analysis of metabolic pathways in *L. chinensis* under salt and alkali stress. (**A**) CK−MS; (**B**) CK−SS; (**C**) CK−MA; (**D**) CK−SA. Each circle in the figure represents a metabolic pathway. Circle color from shallow to deep represents *p*-values from large to small, and circle radius from small to large represents impact value from small to large. The deeper the circle color and the greater the radius, the greater the impact of stress on this pathway. The circle closer to the upper right corner of the pathway diagram represents the more significant effect of stress on this pathway. (1) Glyoxylate and dicarboxylate metabolism; (2) glycine, serine, and threonine metabolism; (3) glyoxylate and dicarboxylate metabolism; (4) TCA cycle; (5) butanoate metabolism; (6) alanine, aspartate, and glutamate metabolism; (7) TCA cycle; (8) glyoxylate and dicarboxylate metabolism; and (9) C5-branched dibasic acid metabolism. CK: control group; MS: 200 mmol/L NaCl treatment; SS: 400 mmol/L NaCl treatment; MA: 25 mmol/L Na_2_CO_3_ treatment; SA: 50 mmol/L Na_2_CO_3_ treatment.

Pathway analysis found that under different concentrations of salt stress and alkali stress, a total of six metabolic pathways were significantly affected. Under mild salt stress, glycine, serine, and threonine metabolism and glyoxylate and dicarboxylic acid metabolism were significantly affected. Under severe salt stress, the glyoxylate and dicarboxylic acid metabolism and the TCA cycle were significantly affected. Under mild alkali stress, butanoate metabolism and alanine, aspartate, and glutamate metabolism were significantly affected. Meanwhile, under severe alkali stress, glyoxylate and dicarboxylic acid metabolism, the TCA cycle, and C5-branched dibasic acid metabolism were significantly affected (Figure 7 and Table 1).

Pathway analysis found that glycine, serine, and threonine metabolism was significantly affected only under mild salt stress, and the content of the intermediate product glycine, which is the main precursor for betaine synthesis, increased significantly. Because this is one of the primary mechanisms for betaine synthesis, the activation of this pathway in response to mild salt stress may be associated with the stimulation of betaine accumulation to participate in osmoregulation [31]. Butanoate metabolism and alanine, aspartate, and glutamate metabolism were only significantly affected under mild alkali stress, and the content of the intermediate product GABA was significantly reduced. GABA, as a non-protein amino acid, is linked to carbon and nitrogen metabolism in plants [32]. Therefore, the significant decrease in its content may be related to its use as a carbon source to promote the significant accumulation of soluble sugars under mild alkali stress, thereby participating in osmotic regulation and energy supply. In previous studies on maize, it was also found that GABA promoted the accumulation of soluble sugar in its seedlings [33]. The C5-branched dibasic acid metabolism was only significantly affected under severe alkali stress, and the content of the intermediate product citraconic acid was significantly increased. The induction of this pathway further emphasizes the important role of organic acid metabolism in *L. chinensis* in response to severe alkali stress; however, the specific physiological functions of butanoate metabolism and C5-branched dibasic acid metabolism in plant stress resistance still need further study.

Pathway analysis also found that glyoxylate and dicarboxylate metabolism was significantly affected under severe salt and alkali stress, and the contents of related intermediates increased significantly, indicating that severe salt and alkali stress activated this pathway in *L. chinensis*. Since this pathway is closely connected with multiple pathways, such as glycine, serine, and threonine metabolism, alanine, aspartate, and glutamate metabolism, the TCA cycle, carbon fixation, and nitrogen metabolism, it also participates in energy transport and provides energy for plants. Therefore, it plays an important role in balancing metabolic disorders and energy deficits caused by stress [34,35]. This pathway was found to be significantly affected in the pathway analysis of *Suaeda salsa* [34], *Bambusa beecheyana* var. *pubescens* [35], and sweet sorghum [36] in response to salt and alkali stress. Combined with the results of promoting the TCA cycle under severe salt and alkali stress, since both pathways are involved in energy metabolism to provide energy for plants, it is speculated that the activation of glyoxylate and dicarboxylate metabolism by *L. chinensis* under severe salt and alkali stress may be related to the promotion of energy metabolism to make up for the energy deficit caused by stress, and it proves that energy supply is one of the important ways for *L. chinensis* to respond to severe salt and alkali stress.

## 3. Materials and Methods

### 3.1. Experimental Materials and Culture Substrates

*L. chinensis* seeds were collected from the artificial *L. chinensis* grassland at Northeast Normal University’s Grassland Ecosystem Field Station (123°44′ E, 44°40′ N, 167 m asl) in Jilin Province, and the experiment was conducted in the greenhouse on the Northeast Normal University campus in Changchun City, Jilin Province. The experiment began on 15 April 2020, with the sand culture approach. First, the cleaned river sand, after sieving, was placed in a seedling tray. We chose seeds that were full-grain and uniform in size. The surface of the seeds was sterilized with 2% sodium hypochlorite for 20 min, rinsed three times with distilled water, dried with filter paper, and placed equally apart on the seedling plate. To ensure enough water supply during the seed germination, we watered the trays with 1 L of distilled water twice a day, at 7:00–8:00 and 17:00–18:00. After the seeds sprouted, we irrigated them once a day at 17:00–18:00 with 0.5× Hoagland nutrient solution; the nutrition solution formula refers to Cao [37]. We chose seedlings with consistent development, a soft green color, and good vitality for transplantation, when the third leaf of the seedling was fully unfolded. The transplant took place on 10 May 2020. Five seedlings were transplanted into plastic flowerpots (30, 30, and 50 cm, with a hole of 2 cm at the bottom and 7.5 kg of river sand), and the distance between the seedlings was kept constant. The pots were placed in the greenhouse for cultivation. The temperature range in the greenhouse during the experiment was 20–28 °C during the day and 15–20 °C at night, and the humidity was 60%.

### 3.2. Stress Treatment

Following transplantation, the seedlings were irrigated with 0.5 Hoagland nutrient solution, 1 L per pot, once every four days (when the sand on the pot’s surface was completely dry), from 17:00 to 18:00, five times in total, for a total of twenty days. The stress treatment started on 1 June 2020. At this time, the *L. chinensis* plants were in the late tillering stage. Based on the results of previous experiments, two concentration gradients, low and high, were designed for salt and alkali stress. The salt stress substance was neutral salt NaCl, mixed in 0.5× Hoagland nutrient solution to make 200 (MS) and 400 mmol·L^−1^ (SS) solutions. The alkali stress substance was Na_2_CO_3_ mixed in 0.5× Hoagland nutrient solution to make 25 (MA) and 50 mmol·L^−1^ (SA) solutions. The control group was continuously treated with the 0.5× Hoagland nutrient solution. The treatment times, cycles, and solution volumes were the same as above. Each pot was regarded as an individual replicate, and each treatment was replicated six times, for a total of 30 pots. A total of 8 treatments were performed for 32 days, and the samples were collected on 2 July 2020, when the *L. chinensis* plants were in the booting stage.

### 3.3. Physiological Indicator Determination Sample Selection and Growth Status Investigation

At the end of the experiment, one plant was chosen at random from each pot, and the third leaf from the top was selected as the material for testing the metabolome. A total of six leaves were instantly frozen in liquid nitrogen and stored at −80 °C for metabolite extraction. Next, another plant was chosen from each pot, and the third leaf from the top was picked separately. A total of six leaves were quickly frozen in liquid nitrogen and stored at −80 °C to determine the content of malondialdehyde (MDA) and relative conductivity. Then, from each pot, another plant was selected, and the third leaf from the top was picked. A total of six leaves were treated in an oven at 105 °C for 15 min, after which the leaves were transferred to a vacuum dryer at 40 °C and dried to a constant weight. Finally, the dry samples were ground with a ball mill and stored for analysis of the amino acids, soluble sugars, betaine, Na^+^, K^+^, and organic acids. Finally, the remaining two plants from each pot, a total of 12 plants, were sampled, washed with distilled water, wiped off the surface moisture, divided into shoots and roots, and the plant heights and root lengths were measured. Then they were dried in an oven at 75 °C to a constant weight, and the shoot dry weight and root dry weight were measured.

### 3.4. Physiological Indicator Determination

The MDA was determined by the thiobarbituric acid method, referring to the method of Zhou [38], and the relative conductivity was determined by a portable DDG-2080-S conductivity meter (DDG-2080-S, Anhui, China). The contents of amino acids, soluble sugar, betaine, and superoxide dismutase (SOD) were determined with ELISA kits (Shanghai Enzyme-linked Biotechnology Co., Ltd., Shanghai, China). The contents of Na^+^ and K^+^ were measured by a DX-300 ion chromatograph (DIONEX Sunnyvale, USA), and the contents of organic acid were determined by ion chromatography (DX-300 ion chromatography system produced by DIONEX, Sunnyvale, CA, USA, with perfluorobutyric acid as the mobile phase), referring to the method of Guo et al. [39].

### 3.5. Metabolite Extraction and Detection

The extraction and detection of metabolites referred to the methods of Kind [40] and Dunn [41]. About 50 mg (49.96 ± 0.19) of the sample was transferred to a 2 mL EP tube, and 0.4 mL of extract solution (methanol/water = 3:1) was added. Then, 20 μL of ribitol and porcelain beads was added, ground at 45 Hz for 4 min, and sonicated for 5 min (ice-water bath). The samples were centrifuged at 4 °C and 13,000 rpm for 15 min, and then 0.2 mL of supernatant from all samples was removed, put into 2 mL injection vials (methane silylated), and mixed with 7 μL of each sample to form a QC sample.

We dried the extract in a vacuum concentrator, added 40 μL of methoxy amination hydrochloride (20 mg/mL in pyridine) to the dried metabolite, gently mixed it, and then incubated it at 80 °C for 30 min in the oven. Then, 60 μL of BSTFA (containing 1% TCMS, *v*/*v*) was rapidly added to each sample, and the mixture was incubated at 70 °C for 2 h. After cooling to room temperature, 10 μL of saturated fatty acid methyl ester standard mixture, dissolved in chloroform (FAMEs, C8-C16: 1 mg/mL; C18-C24: 0.5 mg/mL) was mixed and tested on the machine.

The Agilent 7890 gas chromatography-time-of-flight mass spectrometry instrument was equipped with an Agilent DB-5MS capillary column (30 m × 250 μm × 0.25 μm, J&W Scientific, Folsom, CA, USA), and the specific analysis conditions of GC-TOF/MS were as follows: An aliquot of the analyte (1 μL) was injected in splitless mode; the carrier gas was helium. The front inlet purge flow rate and column flow rate were 3 mL min^−1^ and 1 mL min^−1^, respectively. The column temperature was 50 °C for 1 min, then raised to 310 °C at a rate of 10 °C min^−1^ and kept for 8 min. The front injection port temperature, transfer line temperature, and ion source temperature were 280 °C, 270 °C, and 220 °C, respectively. The ionization voltage was set at −70 eV. The mass spectrometry data were acquired in the full-scan mode with a range of 50–500 *m*/*z* at a rate of 20 spectra per second after a solvent delay of 455 s.

### 3.6. Metabolome Data Processing and Multivariate Data Analysis

Raw data preprocessing included the following steps: Individual data were filtered to remove noise, outliers were filtered based on the interquartile range or relative standard deviation (RSD), and single peaks were filtered; the only peak area data retained had no more than 50% of the null value in a single group or in all groups. A small value of half of the minimum original data was used to simulate the missing value. The data were normalized using the internal standard method (IS).

The preprocessed data were subjected to principal component analysis (PCA) and orthogonal partial least squares discriminant analysis (OPLS-DA) with Simca 14.1 software (Umetrics, Umea, Sweden). When performing PCA analysis, the data were first logarithmically transformed and formatted centrally in the software, followed by automatic modeling analysis. In order to filter out orthogonal variables in metabolites that were not related to the categorical variables and obtain more reliable metabolite information on group differences, OPLS-DA was used to extract the maximization information on the metabolic changes under stress and the importance of metabolites leading to such changes (vip value). Differential metabolites were screened by Student’s *t*-test (*p* < 0.05) and the importance value (vip > 1) and identified according to the Fiehn database (https://fiehnlab.ucdavis.edu/, accessed on 5 October 2020). Differential metabolic pathways were annotated according to the KEGG database (https://www.kegg.jp/kegg/pathway.html, accessed on 20 October 2020). Pathway enrichment analysis was conducted according to Metaboanalyst 5.0 (https://www.metaboanalyst.ca/, accessed on 10 November 2020) to find the key pathways most related to differential metabolites (*p* < 0.05, impact > 0.1).

### 3.7. Statistical Analysis

Statistical analysis was performed using SPSS 20 (SPSS Institute, Cary, NC, USA) for growth parameters, ions, MDA, relative conductivity, soluble sugar, amino acids, betaine, and organic acids. All treatments were repeated six times, and the significant differences between the control and the stress passed the *t*-test.

## 4. Conclusions

*L. chinensis* mainly accumulated soluble sugars and amino acids under mild salt and alkali stress, while it mainly accumulated organic acids under severe salt and alkali stress. As a typical small molecular osmotic regulator, betaine accumulated significantly under different concentrations of salt and alkali stress. In addition to metabolites, there were also differences in the metabolic pathways of *L. chinensis* in response to different concentrations of salt and alkali stress. Under mild salt stress, it mainly induced glycine, serine, and threonine metabolism and promoted the synthesis pathway of betaine. Under mild alkali stress, butanoate metabolism and alanine, aspartate, and glutamate metabolism were mainly induced, and GABA was used as a carbon source to promote the synthesis of soluble sugars. Meanwhile, under severe salt stress and alkali stress, the TCA cycle was mainly activated to provide energy, and at the same time, glyoxylate and dicarboxylic acid metabolism was significantly affected since this pathway is closely connected with the TCA cycle; therefore, activating this pathway to promote energy metabolism and accumulating energy substances may be one of the main ways for *L. chinensis* to respond to severe salt and alkali stress. This study demonstrated that *L. chinensis* responds to and alleviates the damage caused by various types and intensities of salt and alkali stress by accumulating different organic solutes and small-molecule metabolites. It also discovered the importance of betaine in stress resistance as well as the significance of organic acid in severe salt stress, and it further showed that energy supply is one of the key mechanisms in response to severe salt and alkali stress.

## Figures and Tables

**Table 1:** Pathway enrichment analysis depicts significantly changed metabolic pathways in *L. chinensis* in response to salt and alkali stress. CK: control group; MS: 200 mmol/L NaCl treatment; SS: 400 mmol/L NaCl treatment; MA: 25 mmol/L Na_2_CO_3_ treatment; SA: 50 mmol/L Na_2_CO_3_ treatment.

	Pathway	Total	Expected	Hits	*p*-Value	FDR	Impact
CK-MS	Glycine, serine, and threonine metabolism	33	0.0470	1	0.0465	0.6311	0.2118
	Glyoxylate and dicarboxylate metabolism	29	0.0413	1	0.0409	0.6311	0.1015
CK-SS	TCA cycle	20	0.0855	2	0.0028	0.2660	0.1608
	Glyoxylate and dicarboxylate metabolism	29	0.1240	2	0.0059	0.2793	0.1110
CK-MA	Butanoate metabolism	17	0.0364	1	0.0359	0.7970	0.1364
	Alanine, aspartate, and glutamate metabolism	22	0.0470	1	0.0463	0.7970	0.1295
CK-SA	TCA cycle	20	0.1426	3	0.0003	0.0265	0.1939
	Glyoxylate and dicarboxylate metabolism	29	0.2067	3	0.0009	0.0411	0.1685
	C5-Branched dibasic acid metabolism	6	0.0428	1	0.0421	1.0000	0.5000

## Data Availability

Research data are not shared.

## Supplementary Materials

The following supporting information can be downloaded at: https://www.mdpi.com/article/10.3390/plants12091916/s1, Supplementary Table S1: Metabolome raw data of *L. chinensis* under different concentrations of salt and alkali stress; Supplementary Table S2: Contribution of *L. chinensis* metabolites to the first principal component (PC1) and the second principal component (PC2); Supplementary Table S3: Contents and fold change of differential metabolites in *L. chinensis* under salt and alkali stress. The value marked in red indicates that the content of this metabolite changes significantly under stress (*p* < 0.05).

## References

[1] Liu B.S., Hu Y.H., Wang Y., Xue H.H., Li Z.H., Li M. (2022). Effects of saline-alkali stress on bacterial and fungal community diversity in *Leymus chinensis* rhizosphere soil. Environ. Sci. Pollut. R.

[2] Guo R., Yang Z.Z., Li F., Yan C.R., Zhong X.L., Liu Q., Xia X., Li H.R., Zhao L. (2015). Comparative metabolic responses and adaptive strategies of wheat (*Triticum aestivum*) to salt and alkali stress. BMC Plant Biol..

[3] Fang S.M., Hou X., Liang X.L. (2021). Response Mechanisms of Plants Under Saline-Alkali Stress. Front. Plant Sci.

[4] Krasensky J., Jonak C. (2012). Drought, salt, and temperature stress-induced metabolic rearrangements and regulatory networks. J. Exp. Bot..

[5] Khan N., Bano A., Rahman M.A., Rathinasabapathi B., Babar M.A. (2019). UPLC-HRMS-based untargeted metabolic profiling reveals changes in chickpea (*Cicer arietinum*) metabolome following long-term drought stress. Plant Cell Environ..

[6] Guo R., Shi L.X., Yan C.R., Zhong X.L., Gu F.X., Liu Q., Xia X., Li H.R. (2017). Ionomic and metabolic responses to neutral salt or alkaline salt stresses in maize (*Zea mays* L.) seedlings. BMC Plant Biol..

[7] Gao Y.G., Jin Y.L., Guo W., Xue Y.W., Yu L.H. (2022). Metabolic and Physiological Changes in the Roots of Two Oat Cultivars in Response to Complex Saline-Alkali Stress. Front. Plant Sci..

[8] Guo J.X., Lu X.Y., Tao Y.F., Guo H.J., Min W. (2022). Comparative Ionomics and Metabolic Responses and Adaptive Strategies of Cotton to Salt and Alkali Stress. Front. Plant Sci..

[9] Zhang J., Yang D.S., Li M.X., Shi L.X. (2016). Metabolic Profiles Reveal Changes in Wild and Cultivated Soybean Seedling Leaves under Salt Stress. PLoS ONE.

[10] Xiao C.X., Cui X.L., Lu H.Y., Han L., Liu S.B., Zheng Y.X., Wang H., Wang H., Yang C.W. (2020). Comparative adaptive strategies of old and young leaves to alkali-stress in hexaploid wheat. Environ. Exp. Bot..

[11] Yang N., Song X.Q., Lu X.Y., Chen Q., Liu J., Liu Y., Wang H.Z., Zhang Z.H., Tang Z.H. (2021). Comparative study on metabolites and elements of two dominant plant communities in saline-alkali grassland. Environ. Exp. Bot..

[12] Liu B.S., Kang C.L., Wang X., Bao G.Z. (2015). Tolerance mechanisms of *Leymus chinensis* to salt–alkaline stress. Acta Agric. Scand. Sect. B—Soil Plant Sci..

[13] Li J.K., Cui G.W., Hu G.F., Wang M.J., Zhang P., Qin L.G., Shang C., Zhang H.L., Zhu X.C., Qu M.N. (2017). Proteome dynamics and physiological responses to short-term salt stress in *Leymus chinensis* leaves. PLoS ONE.

[14] Wang H., Xiang Y., Li L.H., Bhanbhro N., Yang C.W., Zhang Z. (2020). Photosynthetic response and transcriptomic profiling provide insights into the alkali tolerance of clone halophyte *Leymus chinensis*. Photosynthetica.

[15] Lin J.X., Peng X.Y., Hua X.Y., Sun S.N., Wang Y.N., Yan X.F. (2018). Effects of arbuscular mycorrhizal fungi on *Leymus chinensis* seedlings under salt-alkali stress and nitrogen deposition conditions: From osmotic adjustment and ion balance. RSC Adv..

[16] Kuang W., Wei Z., Liu Y., Dai L., Zhao Y., Zhang Y.Z., Fang B.H. (2021). Effects of Flooding Treatment at Booting Stage on Cadmium Accumulation and Transport Characteristics of Different Rice Varieties. Hunan Agric. Sci..

[17] Zhu J.L., Fan G.C. (2021). Effects of Salt Stress at Different Growth Stages on Yield Traits of Rice. J. Zhejiang Agric. Sci..

[18] Yuan C., Lu A.Q., Zhu L., Xu X. (2019). Comprehensive evaluation of salt tolerance of sweet sorghum at booting stage. Agric. Res. Arid. Areas.

[19] Dinneny J.R. (2015). Traversing organizational scales in plant salt-stress responses. Curr. Opin. Plant Biol..

[20] Chen Y.Y., Li Y.Y., Sun P., Chen G.L., Xin J. (2017). Interactive effects of salt and alkali stresses on growth, physiological responses and nutrient (N, P) removal performance of *Ruppia maritima*. Ecol. Eng..

[21] Guo H.J., Hu Z.Q., Zhang H.M., Min W., Hou Z.N. (2019). Comparative Effects of Salt and Alkali Stress on Antioxidant System in Cotton (*Gossypium Hirsutum* L.) Leaves. Open Chem..

[22] Guo C.Y., Wang X.Z., Chen L., Ma L.N., Wang R.Z. (2015). Physiological and biochemical responses to saline-alkaline stress in two halophytic grass species with different photosynthetic pathways. Photosynthetica.

[23] Liu B.S., Kang C.L., Wang X., Bao G.Z. (2015). Physiological and morphological responses of *Leymus chinensis* to saline-alkali stress. Grassl Sci..

[24] Wang Y.N., Tao S., Hua X.Y., Yu X.Y., Yan X.F., Lin J.X. (2018). Effects of arbuscular mycorrhizal fungi on the growth and physiologicalmetabolism of *Leymus chinensis* under salt-alkali stress. Acta Ecol. Sin..

[25] Yang C.X., Zhao W.N., Wang Y.N., Zhang L., Huang S.C., Lin J.X. (2020). Metabolomics Analysis Reveals the Alkali Tolerance Mechanism in *Puccinellia tenuiflora* Plants Inoculated with Arbuscular Mycorrhizal Fungi. Microorganisms.

[26] Zhang L., Yang C.X. (2018). Enzyme Activities and Free Amino Acids of *Puccinellia tenuiflora*-Arbuscular mycorrhizal Symbiont under Saline-alkali Stress. J. Northeast For. Univ..

[27] Adams P., Thomas J.C., Vernon D.M., Bohnert H.J., Jensen R.G. (1992). Distinct cellular and organismic responses to salt stress. Plant Cell Physiol..

[28] Ramanjulu S., Sudhakar C. (2000). Proline metabolism during dehydration in two mulberry genotypes with contrasting drought tolerance. J. Plant Physiol..

[29] Liang P.X., Tang R., Guo R., Liu J.G. (2022). Effect of mixed salt-alkaline stress on growth and physiological characteristicsin *Cyperus esculentus* L.. J. Arid. Land Res. Environ..

[30] Chen W., Feng C., Guo W., Shi D., Yang C. (2011). Comparative effects of osmotic-, salt- and alkali stress on growth, photosynthesis, and osmotic adjustment of cotton plants. Photosynthetica.

[31] He Y. (2010). Study on the Enhancement of Drought Tolerance of Ryegrass by Introduction of the Glycine-Methylation Biosynthetic Pathway of Glycinebetaine. Ph.D. Thesis.

[32] Mishra V., Gahlowt P., Singh S., Dubey N.K., Singh S.P., Tripathi D.K., Singh V.P. (2023). GABA: A key player of abiotic stress regulation. Plant Signal. Behav..

[33] Wang Y.C., Gu W.R., Meng Y., Xie T.L., Li L.J., Li J., Wei S. (2017). γ-Aminobutyric Acid Imparts Partial Protection from Salt Stress Injury to Maize Seedlings by Improving Photosynthesis and Upregulating Osmoprotectants and Antioxidants. Sci. Rep..

[34] Diao F.W., Dang Z.H., Cui X., Xu J., Jia B.B., Ding S.L., Zhang Z.C., Guo W. (2021). Transcriptomic analysis revealed distinctive modulations of arbuscular mycorrhizal fungi inoculation in halophyte *Suaeda salsa* under moderate salt conditions. Environ. Exp. Bot..

[35] Zheng J.M., Chen S.K., Chen L.G., Hong X.L., He T.Y., Zheng Y.S. (2020). Identification and analysis of differential expression proteins in response to salt stressin the leaves of *Bambusa beecheyana* var. *pubescens* in coastal dunes. J. Fujian Agric. For. Univ..

[36] Dai L.Y., Du J.D., Zhang Y.X., Zhu H.D., Yin K.D. (2017). Proteomics analysis of sweet sorghum in response to soda saline-alkali stress. J. Ecol..

[37] Cao M. (2017). Effects of Saline-Alkali Stress on the Individualand Clonal Growth Traits of *Leymus chinensis*. Ph.D. Thesis.

[38] Zhou W.J., Leul M. (1999). Uniconazole-induced tolerance of rape plants to heat stress in relation to changes in hormonal levels, enzyme activities and lipid peroxidation. Plant Growth Regul..

[39] Guo R. (2009). The Study of Saline Alkaline Tolerant Eco-Physiological Metabolism in Four Gramineae in the Songnen Grasslands. Ph.D. Thesis.

[40] Kind T., Wohlgemuth G., Lee D.Y., Lu Y., Palazoglu M., Shahbaz S., Fiehn O. (2009). FiehnLib: Mass Spectral and Retention Index Libraries for Metabolomics Based on Quadrupole and Time-of-Flight Gas Chromatography/MassSpectrometry. Anal. Chem..

[41] Dunn W.B., Broadhurst D., Begley P., Zelena E., Francis-McIntyre S., Anderson N., Brown M., Knowles J.D., Halsall A., Haselden J.N. (2011). Procedures for large-scale metabolic profiling of serum and plasma using gas chromatography and liquid chromatography coupled to mass spectrometry. Nat. Protoc..

