# Supplementary material for: Differences in Organic Solute and Metabolites of Leymus chinensis in Response to Different Intensities of Salt and Alkali Stress
Source: Plants (Basel). 2023 May 8;12(9):1916. doi: 10.3390/plants12091916 (PMC10181334; doi:10.3390/plants12091916)
Supplement: Supplementary file 1 [file plants-12-01916-s001.zip › Supplementary Table S1.pdf]

Supplementary Table S1 Contribution of *L. chinensis* metabolites to the first principal component (PC1) and the second principal component (PC2).

	**Contribution**	
**Peak**	**PC1**	**PC2**
(2R,3S)-2-hydroxy-3-isopropylbutanedioic acid	0.03	0.03
1,2,4-Benzenetriol	0.01	-0.04
1,2-cyclohexanedione	0.08	0.03
1,3-diaminopropane	0.03	-0.03
1,4-cyclohexanedione	0.00	0.04
1,5-anhydroglucitol	0.00	0.05
11-beta-prostaglandin-F-2-alpha	-0.02	-0.03
1-aminocyclopropanecarboxylic acid	0.03	-0.01
1-hydroxy-2-naphthoic acid	0.04	-0.05
1-indanone	0.01	0.02
1-methyladenosine	0.06	-0.03
1-monopalmitin	0.04	0.03
2,2-dimethylsuccinic acid	0.03	-0.04
2,3-dihydroxypyridine	0.05	-0.02
2,3-dimethylsuccinic acid	0.01	-0.07
2,4-diaminobutyric acid	0.08	-0.06
2,6-diaminopimelic acid	-0.05	-0.02
21-hydroxypregnenolone	0.04	0.04
2-amino-3-methoxybenzoic acid	-0.02	0.02
2-butyne-1,4-diol	-0.05	0.01
2-carboxybenzaldehyde	0.03	-0.03
2-deoxyerythritol	0.06	0.00
2'-deoxyguanosine	0.05	0.05
2-hydroxy-3-isopropylbutanedioic acid	0.01	0.01
2-hydroxypyridine	0.00	0.01
2-hydroxyvaleric acid	0.04	-0.02
2-indanone	-0.03	-0.08
2-ketobutyric acid	0.01	-0.02
2-ketovaleric acid	0.05	0.02
2-methoxyestrone	0.01	-0.02
2-methylfumarate	0.03	-0.05
2-monoolein	0.05	-0.01
2-monopalmitin	0.03	-0.01
3,4-dihydroxybenzoic acid	0.03	0.00
3,4-dihydroxycinnamic acid	-0.02	-0.02
3,6-anhydro-D-galactose	0.05	0.01
3,7,12-trihydroxycoprostane	0.00	-0.06
3-aminoisobutyric acid	0.00	-0.07
3-cyanoalanine	0.00	-0.08
3-hexenedioic acid	0.04	-0.07

3-hydroxybenzaldehyde	0.01	0.03
3-hydroxybenzoic acid	0.00	-0.06
3-hydroxybutyric acid	0.03	0.01
3-hydroxy-L-proline	-0.01	-0.06
3-hydroxynorvaline	0.05	0.05
3-hydroxypropionic acid	0.05	-0.05
3-hydroxypyruvate	0.04	0.02
3-Indolepyruvic acid	-0.03	0.00
3-methylamino-1,2-propanediol	0.05	-0.04
3-methyloxindole	0.05	-0.05
4-aminobutyric acid	0.01	-0.04
4-hydroxy-3-methoxybenzoic acid	-0.01	0.08
4-hydroxybutyrate	-0.03	-0.09
4-hydroxycinnamic acid	0.06	0.03
4-vinylphenol	0.06	-0.05
4-vinylphenol dimer	0.02	0.05
5,6-dimethylbenzimidazole	0.07	-0.04
5-dihydrocortisone	0.03	-0.07
5-methoxytryptamine	-0.03	-0.05
6-phosphogluconic acid	0.00	0.07
adenosine 5-monophosphate	0.00	-0.07
adipamide	0.05	-0.03
alanine	-0.02	0.03
allantoic acid	0.02	0.04
allose	-0.03	0.05
alpha-ketoisocaproic acid	0.05	0.06
aminomalonic acid	-0.05	-0.10
arbutin	-0.06	-0.05
asparagine	0.02	0.04
aspartic acid	-0.01	-0.03
atropine	-0.05	-0.03
benzoic acid	0.07	0.06
beta-alanine	0.02	0.01
beta-mannosylglycerate	0.01	-0.10
caffeic acid	0.01	-0.03
canavanine degr prod	-0.02	-0.06
catechol	-0.01	0.00
cellobiose	0.00	0.00
cellobiotol	0.05	-0.02
cetadiol	-0.05	-0.06
chlorogenic Acid	0.00	0.06
citraconic acid	0.05	-0.03
citric acid	-0.07	-0.03
citrulline	0.06	-0.07

conduritol b epoxide	0.08	0.00
coniferyl alcohol	0.08	0.03
corticosterone	0.00	-0.05
cycloleucine	0.00	0.00
cytidine	0.06	-0.03
cytidine-monophosphate degr prod	-0.01	-0.10
cytosin	0.07	0.01
D-glycerol-1-phosphate	-0.05	0.02
D-glucosamine	0.05	0.08
D-altrose	0.08	0.02
D-arabitol	0.04	0.02
dehydroabietic acid	-0.01	0.07
D-erythronolactone	0.05	0.05
D-erythro-sphingosine	-0.02	-0.02
D-galacturonic acid	0.01	0.00
D-glucoheptose	0.03	0.08
D-glyceric acid	0.06	-0.02
digalacturonic acid	0.08	0.00
digitoxose	0.01	0.04
dihydroxyacetone	0.03	0.04
D-talose	-0.03	-0.05
erythrose	-0.01	-0.03
ethanolamine	0.04	-0.03
ferulic acid	0.02	0.05
fructose	-0.06	0.00
fructose 2,6-biphosphate degr prod	-0.04	-0.03
fumaric acid	0.04	-0.05
galactinol	0.07	-0.03
galactonic acid	0.01	0.04
gentiobiose	0.02	0.02
gentisic acid	-0.05	0.05
glucoheptonic acid	-0.01	-0.07
gluconic acid	0.00	-0.01
gluconic lactone	0.01	-0.02
glucose	0.02	0.01
glucose-1-phosphate	0.07	0.03
glutamic acid	0.02	-0.06
glutamine	0.08	0.03
glutaraldehyde	0.03	-0.01
glutaric Acid	0.00	0.03
glutathione - H2O	0.05	0.00
glycerol	0.03	-0.01
glycine	0.08	0.03
glycolic acid	-0.01	-0.03

guanidinosuccinic acid	0.00	0.01
guanine	0.04	0.04
guanosine	0.02	0.03
hesperitin	-0.04	0.02
hydroquinone	0.06	0.03
hydroxylamine	-0.04	-0.05
inosine	0.02	-0.03
isocitric acid	0.02	-0.05
isoleucine	0.03	0.04
isomaltose	-0.05	0.04
isopropyl-beta-D-thiogalactopyranoside	0.00	0.00
itaconic acid	0.07	-0.01
lactamide	0.02	0.08
lactic acid	-0.07	0.03
lactobionic Acid	0.01	-0.01
lactulose	0.06	-0.01
L-allothreonine	0.02	0.00
L-cysteine	0.06	-0.06
levoglucosan	0.03	0.00
L-homoserine	-0.01	0.01
linoleic acid	0.05	0.00
L-malic acid	0.05	-0.06
loganin	-0.02	0.00
L-threose	0.03	0.03
luteolin	0.08	-0.04
lysine	0.06	0.00
maleamate	0.00	-0.07
maleic acid	-0.02	0.06
maleimide	0.03	-0.01
malonic acid	0.07	-0.06
maltitol	-0.02	-0.06
maltose	-0.07	-0.03
maltotriitol	0.04	-0.03
maltotriose	0.07	-0.06
melatonin	0.02	-0.07
melezitose	0.00	-0.04
melibiose	0.08	0.01
methionine	0.03	-0.05
methyl phosphate	-0.03	-0.06
mucic acid	0.01	-0.06
myo-inositol	0.05	0.05
N-2-fluorenylacetamide	-0.01	-0.03
N-acetyl-beta-D-mannosamine	0.04	0.06
N-acetyl-D-galactosamine	0.03	0.02

N-acetyltryptophan	-0.03	-0.05
naringenin	0.00	-0.04
naringin	-0.03	-0.06
N-carbamylglutamate	0.01	0.01
N-cyclohexylformamide	0.08	-0.03
neohesperidin	0.01	0.02
N-ethylglycine	0.01	-0.01
N-ethylmaleamic acid	0.05	0.00
N-formyl-L-methionine	0.04	0.01
N-methyl-DL-alanine	-0.01	0.05
noradrenaline	0.02	0.01
nornicotine	0.08	-0.06
O-acetylserine	0.01	0.01
o-hydroxyhippuric acid	0.01	0.01
oxalacetic acid	-0.02	-0.02
oxoproline	0.04	-0.03
palatinitol	0.02	-0.04
palatinose	0.01	0.02
palmitic acid	-0.03	0.00
pelargonic acid	0.01	-0.03
phenylalanine	0.03	-0.03
phenylethylamine	0.00	-0.02
phosphate	0.04	0.05
phytosphingosine	-0.04	-0.05
proline	0.02	-0.06
prostaglandin A2	-0.03	-0.04
pyrogallol	0.04	0.02
pyrrole-2-carboxylic Acid	0.01	0.01
pyruvic acid	0.00	0.02
quinic acid	0.06	-0.08
raffinose	-0.03	-0.06
resorcinol	0.03	0.02
ribitol	0.05	-0.04
ribose	0.02	0.05
salicin	-0.07	-0.04
salicylic acid	0.06	0.01
serine	0.00	-0.01
shikimic acid	-0.02	0.05
sorbitol	-0.07	-0.03
stearic acid	0.03	0.00
succinic acid	0.01	-0.05
sucrose	-0.01	0.02
sulfuric acid	0.03	0.01
tartaric acid	0.08	0.02

tartronic acid	0.03	0.05
threitol	0.06	-0.01
threonic acid	0.06	-0.06
threonine	-0.01	0.00
thymidine	0.02	0.01
thymine	0.04	0.03
thymol	0.04	0.04
toluenesulfonic acid	0.03	-0.05
trans-3,5-dimethoxy-4-hydroxycinnamaldehyde	0.00	-0.03
trehalose	-0.05	0.02
trehalose-6-phosphate	-0.07	-0.04
tryptophan	0.02	0.00
tyrosine	0.03	-0.02
uracil	0.01	0.03
uridine	0.06	-0.02
valine	0.02	-0.02
xanthosine	-0.02	0.01
xylose	0.05	-0.02
zymosterol	0.02	0.03
