# Supplementary material for: Differences in Organic Solute and Metabolites of Leymus chinensis in Response to Different Intensities of Salt and Alkali Stress
Source: Plants (Basel). 2023 May 8;12(9):1916. doi: 10.3390/plants12091916 (PMC10181334; doi:10.3390/plants12091916)
Supplement: Supplementary file 1 [file plants-12-01916-s001.zip › Supplementary Table S2.pdf]

Supplementary Table S2 Contents and fold change of differential metabolites in *L. chinensis* under salt and alkali stress. The value marked in red indicates that the content of this metabolite changes significantly under stress ($p < 0.05$).

	Concentration					Fold change			
		Salt stress		Alkali stress					
Peak	CK	MS	SS	MA	SA	$Log^{(MS/CK)}$	$Log^{(SS/CK)}$	$Log^{(MA/CK)}$	$Log^{(AS/CK)}$
GABA	0.05±0.04	0.04±0.03	0.00±0.00	0.00±0.00	0.00±0.00	-0.25	-27.24	-27.20	-27.37
glycine	0.00±0.00	0.20±0.15	0.00±0.00	0.00±0.00	0.00±0.00	29.34	-0.01	-0.15	-0.15
proline	0.00±0.00	0.49±0.09	0.00±0.00	0.16±0.19	0.00±0.00	30.61	-0.01	28.96	-0.15
ethanolamine	0.37±0.08	0.36±0.15	0.77±0.25	0.40±0.08	0.46±0.08	-0.06	1.06	0.12	0.30
mucic acid	0.01±0.00	0.01±0.01	0.00±0.01	0.00±0.00	0.01±0.01	0.30	-0.77	-24.90	0.40
phosphate	0.27±0.20	0.43±0.17	0.47±0.39	0.57±0.10	0.52±0.36	0.68	0.80	1.10	0.96
quinic acid	0.02±0.03	0.01±0.01	0.03±0.03	0.02±0.02	0.08±0.03	-1.38	0.17	-0.31	1.86
citraconic acid	0.07±0.02	0.08±0.02	0.08±0.02	0.09±0.07	0.14±0.01	0.11	0.05	0.24	0.94
citric acid	0.04±0.01	0.04±0.03	0.11±0.04	0.10±0.05	0.13±0.06	0.13	1.51	1.43	1.78
isocitric acid	0.00±0.00	0.06±0.07	0.11±0.08	0.00±0.00	0.12±0.07	27.50	28.40	-0.13	28.53
itaconic acid	0.09±0.06	0.05±0.02	0.09±0.06	0.12±0.10	0.21±0.04	-0.95	0.08	0.49	1.26
L-malic acid	0.14±0.07	0.07±0.02	0.22±0.20	0.21±0.18	0.49±0.21	-0.89	0.68	0.61	1.83
malonic acid	0.05±0.02	0.08±0.06	0.08±0.04	0.07±0.00	0.16±0.06	0.68	0.59	0.40	1.66
lactic acid	0.03±0.04	0.03±0.03	0.11±0.05	0.04±0.02	0.13±0.07	0.02	1.86	0.38	2.07
D-Glucosamine	0.53±0.15	0.42±0.14	0.73±0.24	0.89±0.08	0.66±0.12	-0.33	0.48	0.75	0.33
glycerol	0.46±0.10	0.55±0.24	0.71±0.15	0.57±0.19	0.64±0.09	0.27	0.64	0.32	0.49
isomaltose	0.00±0.00	0.08±0.10	0.00±0.00	0.14±0.02	0.00±0.00	28.06	-0.01	28.82	-0.15
lactulose	0.00±0.00	0.01±0.00	0.02±0.01	0.01±0.00	0.02±0.01	0.45	1.96	1.83	1.93
maltotriose	0.07±0.02	0.06±0.02	0.04±0.03	0.05±0.00	0.02±0.03	-0.20	-0.79	-0.45	-1.68
myo-inositol	0.14±0.09	0.14±0.08	0.21±0.06	0.27±0.05	0.21±0.10	0.08	0.64	0.97	0.64
